# The key role of behaviour in animal camouflage

**DOI:** 10.1111/brv.12438

**Published:** 2018-06-21

**Authors:** Martin Stevens, Graeme D. Ruxton

**Affiliations:** ^1^ Centre for Ecology and Conservation, College of Life and Environmental Sciences University of Exeter, Penryn Campus Penryn, TR10 9FE U.K.; ^2^ School of Biology University of St Andrews St Andrews, KY16 9TH U.K.

**Keywords:** camouflage, behaviour, decision‐making, vision, learning, crypsis, movement

## Abstract

Animal camouflage represents one of the most important ways of preventing (or facilitating) predation. It attracted the attention of the earliest evolutionary biologists, and today remains a focus of investigation in areas ranging from evolutionary ecology, animal decision‐making, optimal strategies, visual psychology, computer science, to materials science. Most work focuses on the role of animal morphology *per se*, and its interactions with the background in affecting detection and recognition. However, the behaviour of organisms is likely to be crucial in affecting camouflage too, through background choice, body orientation and positioning; and strategies of camouflage that require movement. A wealth of potential mechanisms may affect such behaviours, from imprinting and self‐assessment to genetics, and operate at several levels (species, morph, and individual). Over many years there have been numerous studies investigating the role of behaviour in camouflage, but to date, no effort to synthesise these studies and ideas into a coherent framework. Here, we review key work on behaviour and camouflage, highlight the mechanisms involved and implications of behaviour, discuss the importance of this in a changing world, and offer suggestions for addressing the many important gaps in our understanding of this subject.

## I. INTRODUCTION

**Figure 1:**
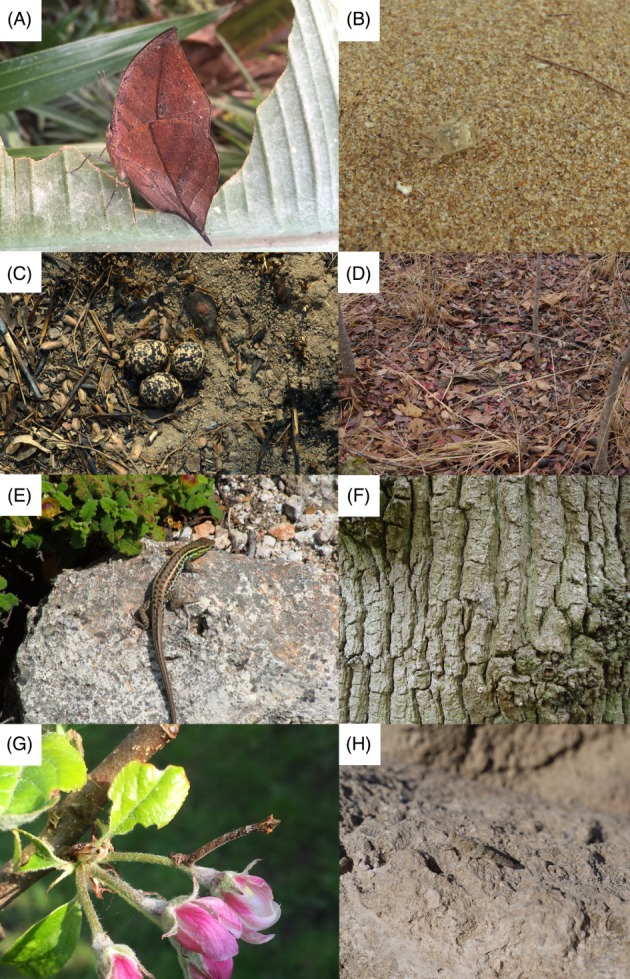
Examples of animals using background choice behaviour for camouflage. (A) *Kallima* leaf‐mimicking butterfly that Wallace noted tends to rest on dead vegetation. (B) Ghost crab (*Ocypode* sp.) which can change colour and selects sand backgrounds that match its individual appearance. (C) Bronze‐winged courser (Rhinoptilus chalcopterus) parents select nesting backgrounds that better match egg appearance. (D) Fiery‐necked nightjars (Caprimulgus pectoralis) select nesting backgrounds to match adult plumage. (E) Aegean wall lizards (*Podarcis erhardii*) choose to sit on backgrounds that match their own appearance better, especially for females and on islands with higher predation rates. (F) Many moths select backgrounds and body positions/orientations to match key features of the background, such as tree bark. (G) Some animals like caterpillars hold their bodies in postures to resemble the shape of twigs and bird droppings. (H) Grasshoppers have been shown to sit in positions that better align themselves with the background, reducing detection.

During his famous voyage in the Malay Archipelago, Wallace was astounded by the camouflage of the Indian leaf mimic butterfly, *Kallima inachus* (Fig. 1A). He noted that part of its resemblance to dead leaves stemmed from its behaviour, habitually resting on dead leaves and twigs, but not on fresh green vegetation (Wallace, 1867). Cott (1940) also noted a variety of cases whereby animals select backgrounds that facilitate camouflage, including observations in ground‐nesting birds, where species nest in locations matching their eggs. Ever since, researchers have noted the importance of background selection in animal camouflage.

In most natural environments, animals face a problem in ensuring that their camouflage is effective – most visual environments vary. This means that a single fixed phenotype is unlikely to be optimally concealed against all or even many potential backgrounds. Three main solutions exist. First, many animals change colour for camouflage, enabling them to tune their appearance to the background (Duarte, Flores & Stevens, 2017a). However, while a few animals such as cephalopods, chameleons, and some fish can rapidly adjust their appearance in seconds (Chiao *et al.,* 2011), colour change in most animals takes longer (Stevens, 2016; Duarte *et al*., 2017a), meaning that there will often be a mismatch between appearance and background during changes. Second, animals might adopt a ‘compromise’ appearance, which matches no background perfectly but several to some degree (e.g. Merilaita, Tuomi & Jormalainen, 1999; Houston, Stevens & Cuthill, 2007), although evidence for how widespread this approach is in nature is lacking. The third solution is for animals to choose where to rest or sit in a way that best matches their appearance (Fig. 1).

**Figure 2:**
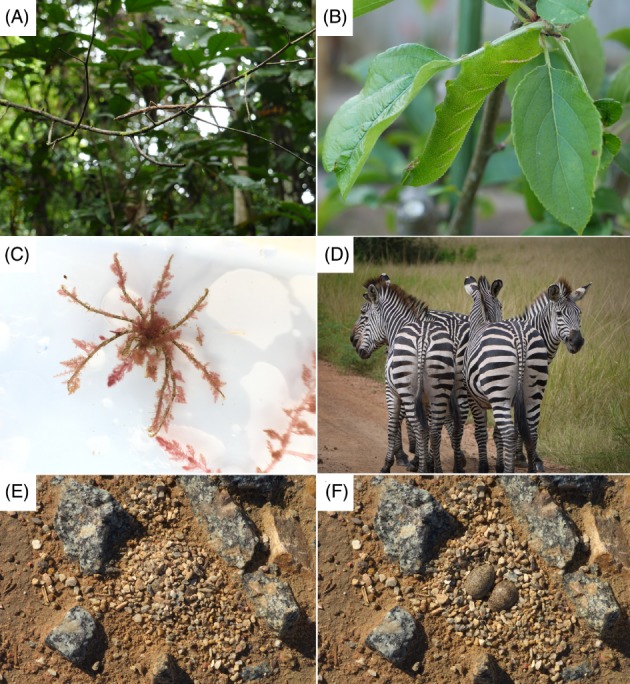
Animals use behaviour in multiple ways to facilitate camouflage. (A) Some insects that mimic twigs or other objects sway in a manner to match background vegetation movement. Species unknown. (B) Animals like this eyed‐hawk moth caterpillar (*Smerinthus ocellata*) orientate their body to facilitate the reduction of shadows. (C) Various species, such as this long‐legged spider crab (Macropodia rostrata) attach material from the environment to their body in decorating behaviour. (D) It has been suggested that the appearance of striped animals, potentially zebra (*Equus* sp.), may cause predators to misdirect attacks due to motion dazzle. (E, F) Some species, such as Kittlitz's plovers (Charadrius pecuarius) modify the nesting environment to hide or camouflage their eggs (E, natural clutch; F, uncovered).

Despite being known for many years, the overall significance of background‐choice behaviour for camouflage has received only sporadic experimental attention and remains largely unappreciated as a key aspect of camouflage. In addition, the role of behaviour in camouflage is much richer than often first appreciated. To begin with, background choice could operate at a species, morph, and individual level; and has a variety of implications for ecology and evolution, from niche partitioning through to the mechanisms controlling behaviour. Regarding the latter, there exists a rich diversity of ways that animals might choose backgrounds for camouflage, and various sensory aspects, and this has implications in fields ranging from non‐human cognition to species responses to anthropogenic change. Behaviour should also play an important role in many other types of concealment, including matching wind‐induced movement in the background, through to camouflage types that operate specifically during movement (e.g. flicker‐fusion camouflage and motion dazzle; Endler, 1978; Stevens, 2007) (Fig. 2; Table 1).

**Table 1:** Summary of the ways that animals can use behaviour to modify and improve camouflage of their own bodies or of related objects (e.g. nests or brood), with selected examples

Behaviour type	Function	Information	Example
*Background choice*	Choose habitat	Animals choose the general habitat in which they are found under which camouflage is improved	Selection of areas by grasshoppers
	Choose patch	Animals choose a specific patch or area within a habitat for camouflage	Morphs of various moth species
	Choose microhabitat	Selection of a highly specific place to rest	Nest site selection by individual ground‐nesting birds
*Body position*	Adopt resting orientation	Specific body positioning and orientation changes to match features of background	Matching of tree bark textures to moth wing patterns
	Change posture	Modify specific body shape or posture to improve efficacy of camouflage	Caterpillars using a bent posture to increase resemblance to bird droppings
	Hide shadows	Hold body in a specific position or angle such that shadows generated on undersides or ground are reduced	Caterpillars with countershading coloration
*Decoration*	Modify own appearance	Attach, coat body in, or trap substances from the environment to change appearance	Decorator crabs attaching corals or algae to legs and carapace
*Modify environment*	Change surroundings	Modify the local surroundings in order to help blending in of own body appearance	Birds creating nest scrapes and adding materials to nests to hide eggs
	Hide objects	Use materials to hide objects (e.g. own body or nest) through improved camouflage	Use of materials in bird nests to camouflage nest structure
	Create decoys	Create or add objects to local environment that resemble the appearance of animal's own body	Orb‐weaving spiders adding detritus and other objects to web that match body appearance
*Movement*	Motion dazzle	Use of high‐contrast markings to prevent accurate estimates of speed and direction by observer	Not well tested but some evidence for zebra stripes partly serving this function
	Flicker‐fusion	During motion the striped markings on animals ‘blur’ and the overall appearance matches the background	Potentially markings on some snakes
	Match environmental motion	Movement that matches the background motion in the environment, such as of wind‐induced vegetation movement	Stick insects swaying with wind

In the last few years, a growing body of research has investigated the role of behaviour in camouflage, and although many gaps remain, it is now appropriate to synthesise what is and is not known regarding this important aspect of anti‐predator coloration. Here, we review and evaluate the role of behaviour in a range of areas related to concealment, from substrate choice through to motion, discuss the potential mechanisms involved, outline considerations for other areas such as conservation, and make a range of suggestions for future work. Herein, we consider camouflage here as strategies that prevent detection or recognition/identification, but also discuss related concepts that involve preventing estimates of movement (e.g. motion dazzle) as these have often been considered forms of camouflage. We focus on visual camouflage, but note that many of the concepts of concealment should apply to non‐visual senses (although they can be hard to differentiate from mimicry and other defences; see Ruxton, 2009). For example, chemical camouflage is likely to occur widely in nature, and when it has been investigated there is often a strong role of dietary choice (see Brooker *et al.,* 2015).

## II. BACKGROUND CHOICE

The most obvious way that animals could use behaviour to improve camouflage is through choosing to rest on backgrounds that match their own appearance (Fig. 1; Table 1). This could arise at a number of levels. First, all individuals of a species may have the same fixed preference for a background type (e.g. all individuals always show a preference for black backgrounds; species‐level choice). Second, all individuals may show the same preferences, but these are context dependent (flexible species‐level choices). For example, when in habitat *a*, all individuals choose white, but all choose black in habitat *b*. Context factors include not just habitat but also, for example, age, activity, reproductive status or level of parasitic infection. In some regards, species‐level choices are expected because many species show preferences for certain (micro)habitats for various reasons not connected to camouflage. It may often be the case that preference for a given background evolves before the camouflage appearance. By contrast, choice could differ among individuals of a species in the same context. Here, individuals of different morphs may choose different backgrounds (e.g. pale individuals choosing white backgrounds and dark individuals choosing black). Finally, choice may be specific to each individual's unique phenotype, especially in slow colour‐changing or highly variable/polymorphic species. These different levels of choice have a variety of implications and would likely be controlled by different mechanisms.

### (1) Evidence of background choice

#### (a) *Species‐level choices*

The majority of early work focussing on species‐level choices tested the behaviour of moths. Sargent (1966) tested background selections of eight species of moth in a box lined with four shades of grey, finding that moths of lighter species selected lighter backgrounds, whereas darker species chose darker backgrounds. Similar results were obtained by Sargent (1968), Sargent & Keiper (1969), Malcolm & Hanks (1973), Boardman, Askew & Cook (1974), and Shreeve (1990). These experiments directly investigated whether moths were more likely to select certain backgrounds, but are limited in that the experimental apparatus was often simplistic and did not represent naturalistic backgrounds, and in some cases moths were tested in groups and could have influenced each other's behaviour. Boardman *et al*. (1974) undertook tests with more natural backgrounds, finding that those species which normally preferred black or white in uniform controlled apparatus also tended to choose dark or light natural substrates, respectively. Fieldwork has furthermore shown preferences of moths for selecting certain backgrounds (Sargent & Keiper, 1969; Endler, 1984). Later work focussed on other taxa. Eterovick & Figueira (1997) studied different species and morphs of grasshopper living in montane fields with visually diverse backgrounds, and investigated choice of substrate when individuals were disturbed. There was a general consistency in the coloration and pattern types of individuals and the substrates that they chose. For example, grey and mottled individuals tended to choose sandy grey soils, whereas individuals with green markings tended to use greener leaves. Unfortunately, because the grasshoppers in this study were grouped into categories based on appearance, rather than true species, it is not possible to determine whether choices are species‐level or morph‐/individual‐specific.

In other work, recently metamorphosed American toads (*Bufo americanus*) show preferences for dark soil and mixed sandy substrates over plain sand, and this coincides with a higher predation risk on plain sand backgrounds from snakes (Heinen, 1993). Some lizards have also been shown to prefer microhabitats that are likely to confer better camouflage. This includes species with preferences for rocky areas over uniform sand and a camouflage strategy that may involve resembling rocks and stones (masquerade; Cooper & Sherbrooke, 2012). Similar results have been found in tortoises (Nafus *et al.,* 2015). Studies manipulating aspects like vegetation cover also report that ground‐nesting birds choose backgrounds related to camouflage and predation risk (Swaisgood *et al.,* 2018; see Section II.1*c*). In birds, choice of perching positions and substrates can also influence camouflage. In species like the common potoo (*Nyctibius griseus*), which subjectively often resemble branches of trees (masquerade), choice of perch sites seems driven for both enhanced background matching and masquerade (Cestari, Gonçalves & Sazima, 2018).

One of the most comprehensive studies to date was undertaken by Kjernsmo & Merilaita (2012) on the least killifish (*Heterandria formosa*), where individuals have a black stripe along their body side. Instead of choosing backgrounds that individuals best match, the authors suggest that prey may instead chose more complex visual backgrounds, since background complexity is known to impede visual detection of targets (Merilaita, 2003; Xiao & Cuthill, 2016). The authors compared choice between differently patterned achromatic backgrounds, controlled to have the same amount of black and white across treatments, but with different arrangement and orientation of stripes and other small shapes. The fish generally preferred backgrounds that allowed better matching with the orientation and shape of their body markings, although in one instance females preferred a more complex background.

#### (b) *Morph‐specific choices*

Many species exist in a number of discrete morphs, and here we would expect background choice to be consistent with morph appearance. Kettlewell (1955) first tested this in an experiment with pale (*typica*) and melanic (*carbonaria*) forms of the peppered moth (*Biston betularia*), and found support for each morph choosing appropriate dark or light backgrounds. Further work by Sargent (1966) and Kettlewell & Conn (1977) with other species is consistent with this, although in some species both pale and melanic forms have been shown to prefer a white background (Sargent, 1969a *;* Lees, 1975), and there is evidence for inconsistent choice among melanic individuals (see Section IV.2; Steward, 1976). Again, the test apparatus in many of these experiments was not very natural in comparison to more recent studies (see Section III.2).

The Pacific tree frog *Pseudacris* (*Hyla*) *regilla* exists in genetic green and brown colour morphs, and Morey (1990) showed that the respective morphs choose substrates according to their appearance. Furthermore, in predation experiments with garter snakes (*Thamnophis elegans*), mismatched frogs were more likely to be attacked than camouflaged individuals. This contrasts with previous experiments on the same colour morphs (Brattstrom & Warren, 1955) that found no evidence of substrate choice. However, later work has shown this species to comprise three morphs: a fixed brown and a fixed green form, and a distinct third morph that can change colour slowly between green and brown (Wente & Phillips, 2003). In behavioural choice experiments (Wente & Phillips, 2005), fixed green frogs preferred matching substrates, but brown morphs only preferred matching substrates when in the presence of predator (snake odour) cues. Surprisingly, colour‐changing individuals showed no preference. It is not clear why such differences in behaviour exist, but different morphs and colour stages may frequent different areas of the canopy and vegetation (Wente & Phillips, 2005).

Isaac & Gregory (2013) studied two geographically separated light and dark colour forms of the western terrestrial garter snake (*Thamnophis elegans*) and used vision modelling to analyse the camouflage of snakes to potential predators (mammals and birds), and their use of basking sites. They measured snake camouflage against resting backgrounds *versus* random samples of nearby backgrounds, and found that snakes consistently chose sites that provided better colour camouflage than expected under random background choice. Thus, on a morph‐specific level at least, snakes seem to make background choices that tune their camouflage. In the polymorphic grasshopper *Circotettix rabula*, which occurs in red and greenish‐grey camouflaged forms, thought to match red granite pebbles and local vegetation respectively, Gillis (1982) also found that grasshoppers selected matching painted backgrounds.

Overall, there is good evidence for background preferences in a range of taxa at a morph‐specific level. However, studies often show that preferences occur in one morph only, or that choices can be inconsistent among individuals. Some species show no clear preferences among morphs at all. A major area to explore is the reasons why such differences among morphs exist, with perhaps some morphs being more specialist and others more generalist for environmental features.

#### (c) *Individual‐level choices*

Variation in appearance within species is frequently not characterised by discrete morphs, but rather by continuous individual variation (although it can be challenging to distinguish the two). Many species show considerable variation among individuals, and this can vary with age and ability to change colour. The ability of individuals to select backgrounds that match their own unique appearance has not been widely considered until recently, except to an extent in colour‐changing species, but several studies have recently tested this idea.

One of the most comprehensive studies of individual background choice was on Japanese quail (*Coturnix japonica*) (Lovell *et al.,* 2013). Quail lay eggs that are highly divergent in appearance among females; some mothers lay light eggs with little maculation, whereas others lay dark eggs with a prominence of dark markings. Lovell *et al*. (2013) gave nesting birds a choice of nesting substrates that ranged in appearance from light sandy colour to dark brown, and then analysed the choices that were made with regard to egg phenotypes. Overall, birds with dark eggs tended to utilise dark substrates, achieving background matching and potential to disrupt the outline of the egg appearance. By contrast, females that laid light‐coloured eggs tended to choose lighter backgrounds, utilising background matching.

Recent evidence from a large‐scale field study shows that wild birds also make refined nest‐site choices that camouflage their egg types better. In a study of nine species of plover, courser, and nightjar from various habitats in Zambia, Stevens *et al*. (2017) showed that wild birds chose backgrounds for nesting that were most appropriate for individual camouflage, to models of predator vision. In the plovers and coursers, parents tend to leave the nest early in response to a threat, leaving the eggs exposed. Background choice in these species was tuned to individual egg appearance. By contrast, nightjars tend to sit tight on their eggs until a threat is close, meaning that the main selection pressure for camouflage is on the adult plumage. Correspondingly, nightjar background choice was based most on improving individual adult camouflage. In addition, Stevens *et al*. (2017) found that substrate choice occurred at several spatial scales: birds chose appropriate sites compared to suitable backgrounds selected by other conspecifics, and not only selected suitable backgrounds in a general nesting area, but also at the level of small‐scale patches within that locality to nest. As such, background selection for camouflage was highly tuned to both the main object under selection for camouflage (adult or egg), and to the spatial scale of the habitat. In moths, which can at least select background type on a species‐ and morph‐specific level, there is also evidence for fine‐scale habitat selection by individuals (see Section III.1).

In lizards, background choice seems linked to both individual appearance and predation risk. Marshall, Philpot & Stevens (2016) showed that in Aegean wall lizards (*Podarcis erhardii*), individuals were found on chosen backgrounds that resembled their own individual dorsal coloration (to predator vision models) better than would be the case if found in locations chosen by other individuals. In addition, this effect was stronger in females, which do not have such prominent sexual markings, and on islands where predation risk was higher (and presumably the need for effective camouflage greater). All this points to lizards behaviourally choosing resting sites on the basis of their own appearance to facilitate camouflage.

A range of work has also been undertaken in tadpoles, where substrate choice can be linked to appearance and aspects of the local environment, such as shelter availability. For example, tadpoles of the tree frog *Bokermannohyla alvarengai* subjectively resemble lichens and the stream substrates and face a variety of predators from within and outside the water. Eterovick, Oliveira & Tattersall (2010) analysed the background choice of free‐ranging tadpoles before and after disturbance. When disturbed, tadpoles tended to move to backgrounds that more closely resembled their brightness and colour, thus improving their camouflage. The exception was that individuals already close to shelter (leaves, rocks, and other refuges) often chose to escape to cover. More recent work on the tadpole *Ololygon machadoi* shows that individuals prefer light‐yellow backgrounds over dark or blue ones, possibly as this facilitates disruptive camouflage with their dark bodies and yellow barred markings (Eterovick *et al.,* 2018). However, this seems to be a species‐level choice and the situation in other species is less clear. For example, in some species there is a lack of evidence of clear substrate preferences, despite differences in predation risk with background types (Espanha, de Vasconcelos & Eterovick, 2016). Other species tend to show general species preferences for dark backgrounds (Ximenez *et al.,* 2012).

Background choice could also benefit animals that utilise other forms of camouflage, such as masquerade. Here, background choice occurs in caterpillars of the early thorn moth *Selenia dentaria*, which resemble twigs (Skelhorn & Ruxton, 2012). When given a choice of microhabitats of different twig sizes, caterpillars select the background with twigs that match their own individual body size better. The strength of preference also changes with growth, in that larger caterpillars show stronger choice, potentially as they are inherently more at risk of detection.

#### (d) *Colour‐changing species*

Differences in individual appearance frequently arise due to the ability of some animals to change colour (Duarte *et al.,* 2017a). Here, we would expect that individuals of species that can change colour fairly slowly show background choice (with choice changing to maximise camouflage as the animal changes colour). By contrast, in animals with rapid colour change there may be no need for substrate choice since they can match many backgrounds quickly (subject to constraints regarding the degree of match possible). The available evidence broadly supports this prediction, but not for all species or individuals. For example, work on cuttlefish (*Sepia officinalis*) has tended not to reveal strong preferences for substrate appearance (Allen *et al.,* 2010), although one study on *Sepia pharaonis* (Lee, Yan & Chiao, 2012) found that cuttlefish raised in an environment with enriched visual information including light and dark rocks and artificial algae showed greater/earlier preferences for high‐contrast patterned backgrounds than individuals reared in environments of plain grey or checkerboard patterns. In fish, there is some limited evidence of substrate choice linked to the ability to change colour (Magellan & Swartz, 2013; Tyrie *et al.,* 2015).

As with work on morph‐specific choices, there is often an asymmetry in colour‐changing species as to when choice actually seems to occur. In slower‐changing fish species, individual flatfish that have been on light backgrounds (for 4–6 weeks) prefer light substrates, whereas dark‐background‐acclimated fish showed no preference (Ryer *et al.,* 2008). In this and other examples it is hard to know why preference occurs only in one subset of individuals. No analysis of camouflage or coloration was undertaken, so it is possible that dark‐acclimated fish did not closely resemble the darker backgrounds. In the filefish *Monacanthus chinensis*, which can switch from a brown to green variant, substrate choice also occurs only in brown individuals (Gilby *et al.,* 2015). Similar findings were reported for larval newts (*Lissotriton boscai*), where lighter larvae that had been on light backgrounds spent more time on light than on dark backgrounds, but darker larvae showed no clear preference (Polo‐Cavia & Gomez‐Mestre, 2017). In crab spiders, substrate (flower colour) preference exists, but only by yellow and not by white individuals, potentially due to different prey‐capture strategies (Heiling *et al.,* 2005). Ghost crabs, *Ocypode* spp. can also adjust their coloration and brightness over hours to match the beach environment better (Stevens, Pei Rong & Todd, 2013). Recent work by Uy *et al*. (2017) showed that lighter and darker individual pallid ghost crabs (*O. pallidula*) choose light and dark sand, respectively, in behavioural trials, whereas intermediate crabs show no preference.

Behavioural choice in individuals may be tuned to the prevailing risk of predation. Many species of grasshopper are capable of colour change over days and weeks, and this can improve background matching (Edelaar *et al.,* 2017). Baños‐Villalba, Quevedo & Edelaar (2018) studied microhabitat choice in azure sand grasshoppers (*Sphingonotus azurescens*) living in an urban habitat – street pavements. They found that individual grasshoppers tended to choose to sit in the spaces between paving stones, and in a detection experiment with human subjects this tends to decrease detection rates. However, interestingly, grasshoppers with worse individual camouflage relative to the pavement were more likely to sit between stones. Furthermore, grasshoppers showed threat‐dependent behaviour in line with their camouflage level, in that individuals with better camouflage allowed predators to come closer before escaping. Similar findings have been found in ground‐nesting birds, whereby flight‐initiation distances from a nest when a threat approaches vary with levels of egg or adult camouflage (Wilson‐Aggarwal *et al.,* 2016).

### (2) Ecological context of choice

Background‐choice behaviour of animals should have a range of important outcomes and implications for exploitation of resources and (micro‐)habitats. In the first instance, a range of camouflaged animals show phenotype–environment associations, which can lead to matching to specific habitats (Stevens *et al.,* 2015; Xiao *et al.,* 2016). Such associations and matching can arise through genetic adaptation (e.g. Rosenblum *et al.,* 2010) or phenotypic plasticity and ontogenetic colour change (Todd *et al.,* 2006; Stevens, Lown & Wood, 2014). However, even in phenotypically plastic species, such as shore crabs (*Carcinus maenas*), which are extremely variable in colour and pattern, background choice is also likely to explain associations at the microscale. For example, Todd *et al*. (2012) and Nokelainen *et al*. (2017) examined associations between crab appearance and substrate/habitat type at a range of spatial scales. In addition to macro‐ and meso‐scale associations, they found that aspects of crab appearance were often associated with specific substrates at a micro (<1 m^2^) scale. The most plausible explanation for this is that crabs behaviourally choose substrates to associate with based on appearance. Indirect evidence of microhabitat selection for camouflage also exists from field studies of freshwater turtles (Xiao *et al.,* 2016). Beyond this, many crabs and other crustaceans show size‐/age‐dependent shifts in habitat use that correlate with changes in appearance (usually through ontogeny), and one potential explanation is that this is related to differences in predation risk and camouflage in different habitats (Todd, Qiu & Chong, 2009; Duarte *et al.,* 2017a).

Behaviour and camouflage type can also be linked to habitat use in other ways. For example, the prawn *Hippolyte obliquimanus* exists in two main morphs – a homogenous type that uses background matching to resemble different algal backgrounds, and a striped form that seems to rely on transparency for concealment (Duarte & Flores, 2017). The homogenous types are able to change colour between algal background species, and stick closely to their matching substrate, whereas the transparent form shows less background affinity and more mobile behaviour and associated morphology (Duarte, Stevens & Flores, 2016; Duarte & Flores, 2017). Transparent prawns are mainly males (and homogenous forms are mostly female), and it is possible that transparent individuals use a type of camouflage less restricted to one background type to afford a more mobile life history and generalist habitat use, including mate‐searching behaviour (Duarte & Flores, 2017). Differences in behaviour and camouflage strategies among morphs also seem to reflect some degree of niche partitioning and responses to spatial and seasonal changes in resources (Duarte *et al.,* 2017b).

## III. ORIENTATION, POSTURE, SHAPE, AND HIDING SHADOWS

Behaviour can be used to fine‐tune camouflage by controlling the orientation, posture and shape of the organism.

### (1) Orientation and positioning behaviour

The pioneering work on animal orientation and crypsis focussed on moths, with evidence of non‐random orientation (Sargent & Keiper, 1969; Sargent, 1969c; Endler, 1984; Grant & Howlett, 1988) with respect to features of the background (e.g. ridges on tree bark) in ways that make detection more challenging in behavioural trials with birds searching in photographic slides for moths (Pietrewicz & Kamil, 1977). More recent work by Webster *et al*. (2009) also found evidence that some species of *Catocala* moth have consistent orientation behaviour, whereas other species of moth do not. They presented human subjects with images of moths superimposed on images of trees with changed orientation, and found that orientation was a key attribute contributing to detection. In addition, when they rotated the tree bark images horizontally, the optimal orientation changed accordingly, showing that the effect of orientation on detection was not due to the moth position *per se*, but rather its interaction with background features. Overall, moths in positions whereby their body markings match the orientation of prominent background features were detected less often than those that mismatched, and this corresponded generally with natural resting behaviour in the field.

The above work was restricted to species‐level implications and choice, rather than individual‐specific behaviour, and was largely based on humans (or computer programmes) positioning moths on backgrounds, which neglects potential fine‐tuning of positioning behaviour that many moths may show. A series of recent studies comprehensively investigated the roles of background selection and positioning in camouflage. First, Kang *et al*. (2012) investigated two species of moth (*Hypomecis roboraria* and *Jankowskia fuscaria*). They collected moths at night and released them in the morning and observed changes between initial landing position and subsequent repositioning behaviour. Individuals frequently moved position and specific background patch to rest on within a few minutes of landing. In addition, when images of moths before and after repositioning were presented to human observers, repositioning behaviour significantly reduced the likelihood of detection. The results were due both to the selection of a new microhabitat patch and to body orientation. Kang *et al*. (2013b) then explored why some moths of *H. roboraria* reposition themselves after landing but other individuals do not. They again used a human detection task to evaluate levels of camouflage and found that repositioning behaviour was related to how effectively camouflaged an individual was on first landing – those that were initially well concealed were less likely to reposition themselves than individuals that landed in orientations and positions that rendered them more visible. This demonstrates individual‐level behaviour tuned to increase camouflage, and that moths must have some way of evaluating their current levels of camouflage (see Section IV.2). These experiments involved real resting backgrounds and conditions, compared to most earlier studies on moth background choice, potentially allowing the moths to behave more naturally. There was also a greater focus on the details of individual behaviour. One possible criticism, however, is that moths were released in the morning, which may have been later than they would normally come to rest.

A limitation of the above two studies is that assessment of camouflage was based on human perception, and that camouflage patterns were not quantified. Kang *et al*. (2015) addressed this by analysing the appearance of *H. roboraria* and *J. fuscaria* using models of avian predator vision and discrimination and image analysis. They also investigated whether repositioning behaviour in these two species was centred around improving background matching or disruptive coloration, using a model of edge detection that evaluates the extent to which prey markings break up body edges. Their results varied to some extent among species, but overall moths improved their camouflage for colour matching, brightness matching, disruptive coloration, and pattern direction in one or both species. Thus, potentially like quail (Lovell *et al.,* 2013), moths may utilise behavioural choice and positioning behaviour to improve both background matching and disruption.

Experiments with artificial moth‐like targets have also provided support for the importance of orientation behaviour and body markings in avoiding detection. Wang & Schaefer (2012) used a widely adopted technique of presenting triangular targets matching background tree bark to wild birds to test survival rates of different prey phenotypes. They created targets with either horizontal or vertical camouflage markings (a single stripe) and pinned them in either a horizontal or vertical orientation. Detection was strongly influenced by the interaction between target marking orientation and position, such that targets with vertical stripes survived better when resting horizontally on bark, whereas targets with horizontal stripes survived better when positioned with the ‘wings’ vertically positioned on the trees. Thus, detection rates were lower when the body stripes coincided with the prominent orientation of the bark structures.

### (2) Posture, shape, and minimising shadows

The positions that animals adopt can also affect types of camouflage beyond crypsis, such as masquerade. Suzuki & Sakurai (2015) noted that many caterpillars which are thought to resemble bird droppings rest in a posture with their bodies curled up or bent, seemingly increasing their similarity to real droppings. They created artificial caterpillar prey that were either black and white or green, and pinned them either in a straight posture or bent, and measured predation from birds in the field. Models that were green (cryptic) did not differ in survival with posture, whereas black‐and‐white models resembling bird droppings survived better when placed in a bent posture. An alternative explanation is that the repeating black and white stripes on the straight models may have enhanced detection compared to those that were bent, where repeating pattern elements are less apparent, and potentially created disruptive benefits. Further work would usefully undertake comparative studies of whether bent postures in insect larvae are strongly associated with masquerade as opposed to other forms of crypsis, and use morphometric analyses to assess how much this improves the match to putative models. Other animals like lizards have been suggested to flatten their bodies, potentially to increase their resemblance to objects like rocks (Cooper & Sherbrooke, 2012).

Another type of camouflage well known to prevent detection is self‐shadow concealment and obliterative shading, *via* countershading coloration (Thayer, 1909). Here, many animals are darker on the surfaces of their body that face higher light intensity, to blend with shadows on the undersides of the body and destroy three‐dimensional shape information. Countershading has been demonstrated to effectively reduce detection of model prey (e.g. Rowland *et al.,* 2007), and detailed modelling of light conditions and predation experiments have shown that the value of countershading is strongly linked to the specific coloration of the animal and the prevailing light conditions and intensity (Cuthill *et al.,* 2016). Given this, animals with countershading patterns should adopt body positions that best place their bodies with regard to light intensity and direction. On a broad level, many animals that rest upside down, such as some caterpillar larvae, are reverse countershaded (darker on their ventral surface). Otherwise, modelling predicts that body orientation should affect the efficacy of countershading for camouflage (Penacchio *et al.,* 2015). However, at present, work on countershading and camouflage is heavily based on artificial systems (mostly modelling of light intensity and body shape, and predation experiments with artificial prey), and future work should explore behavioural aspects of countershading in animals.

## IV. MECHANISMS OF CHOICE AND ORIENTATION

Here we focus both on the senses that animals use to control their behaviour, and the underlying mechanisms that govern how information from the senses is used to control behaviour: whether fixed genetic preference, imprinting, assessment of own camouflage, or otherwise.

### (1) Use of different sensory modalities to judge the background

A range of sensory information can be used to find suitable backgrounds and resting sites. For example, some shrimp use visual information on shape, size, and contrast to locate preferred habitats (Barry, 1974). Gillis (1982) studied substrate choice in the polymorphic grasshopper *Circotettix rabula* (see Section II.1*d*), and found that when the eyes of individual grasshoppers were damaged, substrate choice disappeared. Again, most work has been undertaken in moths, with early studies showing that both visual and texture cues may be important (Sargent & Keiper, 1969; Sargent, 1969c, 1973; Lees, 1975). Kang *et al*. (2013a) tested how moths respond to background cues in positioning behaviour on trees and found support for moths mainly using tactile information related to bark furrow structure. When allowed to choose resting positions on photographs of tree bark, moths orientated without any clear preference or choice. However, when presented with backgrounds made of cardboard with different structures, moths orientated themselves according to the direction of the structures. In general, moths tended to position themselves with their head touching a furrow, which matches their body patterns to the bark structures.

Tests of the sensory information used in background choice can be achieved by varying the available information, as above, or alternatively by disabling each sensory system in turn. Kang *et al*. (2014) undertook such experiments in *J. fuscaria*, presenting them with structured backgrounds made from cardboard in a box. They then investigated choice in the absence of chemical cues (by removing the ends of the antennae), tactile cues (removing the front legs), and visual cues (conducting trials in darkness). When the antennae and front legs were removed, moths still orientated correctly. By contrast, in darkness, moth orientation was inconsistent with regard to background structures. This suggests that visual information is used in orientation behaviour. However, there was some evidence that removing parts of the wings affected the degree of choice, while removing only the forelegs still allowed considerable tactile information to be gathered from the other legs. Thus it remains uncertain to what extent different sensory information is used in guiding background choice and repositioning behaviour. It is interesting that in some cases behavioural choice for a visual defence may be guided by other sensory information (e.g. texture). In other organisms, olfactory information may be particularly important, especially when it is associated with specific background substrate types. Even for vision, studies have yet to link the presence of background choice properly to colour, luminance, and polarisation abilities of the animal in discriminating between potential sites. Overall, much work remains to be done in understanding the sensory basis of background choice and positioning.

### (2) How animals could choose correct backgrounds

One of the most important issues regarding background choice and positioning concerns the mechanisms that enable animals to make appropriate choices. There are several potential ways this could be achieved (Table 2). First, there may exist a genetic basis for choice (‘preference gene’), linked to genes governing appearance. Second, animals could imprint on or learn about certain visual backgrounds that are important or that they are likely to associate with. Third, individuals may actively use their senses to assess how closely their body coloration matches the backgrounds that they choose. Note that these mechanisms are not mutually exclusive, and the mechanisms will likely differ depending on the level of choice (species, morph, individual).

**Table 2:** Evidence for and against the main hypotheses for the mechanisms controlling background‐choice behaviour for camouflage

Mechanism of choice	Evidence for	Evidence against
***Genetic control***: choice of backgrounds is controlled by ‘preference gene’ linked to gene(s) for appearance	Not directly tested, but some evidence in moths with discrete morphs (Steward, 1976, 1977; Grant & Howlett, 1988); indirect evidence from butterfly appearance and mate preference genes (see Section IV.2); most likely in species with fixed appearance and/or little opportunity to learn	
***Contrast‐conflict***: individuals could use vision to compare directly how closely their appearance matches that of the current background	Direct evidence in grasshoppers (Gillis, 1982) and indirect support in fine‐positioning behaviour in moths (Kang *et al.,* 2012, 2013b); most likely in species with relatively quick ability to change coloration plastically and/or with frequent movement to new patches; may control fine‐scale positioning behaviour	Lack of support in direct experiments testing background choice in moths (Sargent, 1968; Grant & Howlett, 1988)
***Imprinting/learning***: animals may imprint over time on the background most likely to provide camouflage or learn to associate features of the background with appearance	Some evidence of modification of choice in cuttlefish (Lee *et al*., 2012) and larval newts (Polo‐Cavia & Gomez‐Mestre, 2017). Indirect support in birds (Pike, 2011); likely to occur in species with slow colour change or fixed ontogenetic changes in appearance, and species that have the opportunity to learn over time about the background	Lack of evidence from basic experiments with moths (Sargent, 1969b *;* Grant & Howlett, 1988)

Kettlewell (1955) suggested that in the case of moths, decisions were potentially made on the basis of determining the contrast between the body coloration around the eyes and that of the tree trunk (a ‘contrast‐conflict’ mechanism). This would seem to be potentially valuable because new variants would be pre‐adapted to make appropriate choices without genetic preferences also having to evolve (Grant & Howlett, 1988). However, in moths, the evidence does not support this mechanism, with experiments modifying moth appearance generally not affecting choice (Sargent, 1968; Grant & Howlett, 1988). Instead, there exists variation in choice among individuals of the same morph across families (parentage) in some moths, which is indirectly consistent with genetic control of choice (Steward, 1976, 1977; Grant & Howlett, 1988). This is consistent too with other findings, such as appearance and mate choice in *Heliconius* butterflies, which has shown genetic linkage between coloration and preference genes (Kronforst *et al.,* 2006; Merrill *et al.,* 2011). While much previous work focussed on species‐ and morph‐specific choices, Kang *et al*. (2012, 2013b) showed that individual moths can adjust their specific resting sites and orientations, and that they do this in line with their current levels of camouflage. Thus, there must be mechanisms in moths that facilitate more flexible behavioural strategies. It may be that broad‐scale preferences are under genetic control, whereas fine‐scale decisions require some sort of self‐assessment. Generally in moths, there is no support for imprinting or experience affecting choice (Sargent, 1969b *;* Grant & Howlett, 1988), albeit with very limited testing.

The strongest evidence in support of the ‘contrast‐conflict’ mechanism comes not from moths but from the grasshopper *Circotettix rabula*. Gillis (1982) added a painted mask around the eyes of different grasshopper morphs and, in contrast to moths, found considerable changes in colour preference. When red individuals had a green mask added they switched their preference from red to green backgrounds. Adding a red mask to green individuals abolished any preference. Adding masks of their original colour did not affect grasshopper behaviour, demonstrating that the observed results were not due to the manipulation itself. Finally, unchanged red individuals continued to prefer red, while red individuals with a painted blue mask (an ecologically less relevant colour) now preferred to rest on a blue background. Overall, this provides strong evidence for grasshopper choice based on a contrast‐conflict mechanism. An interesting avenue for future work will be to explore how choices are made in terms of colour and luminance (lightness) visual mechanisms.

Gillis (1982) suggests that a contrast‐conflict mechanism is likely to be especially important in species that undergo ontogenetic changes in colour (which applies to many species; Todd *et al*., 2009), whereby a fixed genetic preference would become maladaptive. Ontogenetic changes are common in many grasshoppers. Indeed, we would expect this to be true in any species that change colour because preferences should be flexible enough to match current coloration. Thus, we predict that in species with a fixed appearance, preferences may be either fixed and controlled by a preference gene, or learnt during key life stages like breeding in birds and egg coloration, whereas in species that undergo plastic or ontogenetic changes the mechanism should be contrast‐conflict or continuous imprinting and re‐imprinting.

In the Polo‐Cavia & Gomez‐Mestre (2017) study of larval newts, preferences for backgrounds that matched their body lightness better could either be explained by imprinting over a period of days on the preferred background, or by a comparison between body and background coloration by the newt. Without experiments preventing newts from seeing their own appearance the two cannot be disentangled. Likewise, work on ghost crabs by Uy *et al*. (2017) showed that darker and lighter crabs preferred darker and lighter backgrounds, respectively. However, these crabs were also much more likely to originate from darker and lighter sandy parts of the beach, respectively, meaning that they could have imprinted on their original background. Work on juvenile *Sepia pharaonis* (Lee *et al*., 2012) also shows a strong effect of rearing conditions on background‐choice behaviour, and showed that exposure to visual information can change preference behaviour. Note that in all these examples, the animals are capable of colour‐pattern change, and so imprinting or experience of substrates may be more likely to be present in such animals.

In ground‐nesting birds like quail and plovers, which show background choice of nesting sites based on egg appearance (Lovell *et al.,* 2013; Stevens *et al.,* 2017), it should be possible to tease apart whether this is innate (genetic) or learnt, by comparing the substrate preferences of first‐time *versus* experienced mothers that have had the opportunity to see their own eggs. Indeed, it has been shown that quail can learn to recognise egg appearance (Pike, 2011). In hosts of avian brood parasites, first‐time breeders need to learn and imprint on their own egg phenotypes and then reject eggs in the nest that deviate from this learnt appearance (Rothstein, 1975).

## V. DECORATION AND BACKGROUND MODIFICATION

Animals interact behaviourally with the world around them, and so now we focus on aspects of this interaction related to the exploitation of materials from the environment (or secreted by the animal itself) to influence their camouflage (Fig. 2).

### (1) Decoration

Ruxton & Stevens (2015, p. 2) reviewed the literature on decorating by animals, and define a decorator as ‘an organism that (by means of specialist behaviour and/or morphology that has been favoured by selection for that purpose) accumulates and retains environmental material that becomes attached to the exterior of the decorator’. For our purposes it is important to note in this definition that decoration can involve behaviour but need not. It is also important to note that the definition is agnostic with respect to function – and while camouflage is one possible function of decoration, numerous others have been postulated.

The most widely studied group of decorators are crabs of the superfamily Majoidea – which has over 900 species of which over 75% show decorating behaviour – with environmental materials selected and placed using the limbs on hooks present over at least part of their bodies. The consensus in the literature is that decorating provides camouflage (through some combination of background matching, disruption and masquerade), but the main evidence for this is elimination of other plausible functions rather than positive evidence for camouflage (Hultgren & Stachowicz, 2008). The strongest line of circumstantial evidence is that decoration is concentrated over areas of the rostrum that would otherwise increase the visibility of these largely sedentary animals to predators, for example, concealing movement of the antennae; decoration is not sufficiently extensive across the body to provide effective physical protection from predators. Hultgren & Stachowicz (2008) also demonstrated that tethered crabs with decorations removed were more likely to be attacked than those in a control group with their decorations intact, presumably mainly *via* a camouflage effect. They also note that crab species that were able to change colour at moult, so as to match their current substrate better, exhibited lower use of decorating, further supporting decorating functioning as a camouflaging behaviour. Recently, Brooker *et al*. (2017) demonstrated that provision of shelters to captive crabs reduced their propensity to decorate – suggesting a cost to decoration that may make it a less‐attractive option when an alternative anti‐predatory behaviour (fleeing to a shelter) is available. They point out that the visual camouflage may have elements of background matching, or disruptive camouflage and of masquerade – but no study has so far explored how (if at all) camouflage is achieved through decoration.

Larvae of a range of insects behaviourally construct shields that involve environmental materials stuck together and to the animal by faecal material. There is evidence that such ‘trash packages’ of juvenile lacewings of several species deter attacks from predators – with the authors (Hayashi & Nomura, 2011) inferring that this stems from failure to detect, localise, and recognise the decorator. A similar situation has been reported with respect to two layers of decoration often sported by assassin bugs (a ‘dust coat’ of dust, sand and soil; and a ‘backpack’ of prey corpses attached to their back with faecal material; Brandt & Mahsberg, 2002).

In birds, a range of species add substances to their feathers that alter their appearance [termed ‘cosmetic coloration’ and reviewed by Delhey, Peters & Kempenaers, 2007]. In most cases these are self‐secreted preen oils, but in some cases they are environmental substances. Staining of the feathers with soil has been observed in a number of large birds and has universally been attributed to camouflage. However, it has been most carefully studied in the rock ptarmigan (*Lapogus mutus*). Both sexes sport all‐white plumage at the start of the breeding season, as snow melts this becomes conspicuous and females moult to produce brown patterned feathers that appear to offer good camouflage. By contrast, males do not moult immediately but smear their feathers with soil before later moulting into a brown plumage. Montgomerie, Lyon & Holder (2001) argue that the plumage soiling is unlikely to be a non‐functional side effect of dust‐bathing since many birds dust‐bathe without noticeable long‐term soiling of their plumage. The responses of females, other males, or predators to immaculate white *versus* soiled plumage has yet to be explored, nor is it clear why the behaviour is restricted to males.

Mayani‐Parás *et al*. (2015) convincingly demonstrate that the blue footed booby (*Sula nebouxii*) has evolved a specialist behaviour in which it rolls its eggs around in the substrate of the nest scrape in such a way that soil adheres to the shell in sufficient quantities to substantially change their appearance and make them more similar in appearance to the surrounding substrate. An *in situ* experiment with chicken eggs demonstrated that such soiling reduced the vulnerability of eggs to gulls. The authors speculated that this form of camouflage through behavioural background matching is a form of phenotypic plasticity that allows the boobies to take advantage of diverse potential breeding substrates. They speculate that this might be particularly important for the blue‐footed booby where competition for nesting sites can be intense and thus individuals can be forced into breeding on non‐preferred substrates. Holveck *et al*. (2017) studied the appearance of the eggs of black‐legged kittiwakes (*Rissa tridactyla*) and argued that soiling could reduce long‐range detection by potential avian predators without obscuring information available to conspecifics (e.g. about maternal quality) at shorter range from the pigment patterns causing speckling.

In summary, decorating behaviour occurs across diverse animal groups. Specialised behaviours are often involved, and camouflage is a likely functional explanation supported by circumstantial evidence. However, plausible alternative explanations often exist – and we currently lack a definitive study that tests for decoration conferring an anti‐predatory advantage under wild conditions through enhanced camouflage.

### (2) Modifying the visual background

We have already considered how animals can influence camouflage behaviourally by selection of the background against which they are seen. It seems at least conceptually plausible that animals could go a step further and behaviourally modify that environment to enhance crypsis.

Troscianko *et al*. (2016) investigated the egg‐covering behaviour of Kittlitz's plover (*Charadrius pecuarius*). Many birds cover their eggs prior to leaving the nest, but the reason for this is often considered to be thermoregulatory. However, in Kittlitz's plover, egg covering with stones and vegetation does not occur unless there is an immediate predatory threat, and eggs are not entirely covered. Troscianko *et al*. (2016) demonstrated that the material selected for use in egg‐covering provided a close match to the coloration of the eggs but not to the wider background substrate. Hence the authors interpret the possible selective benefit of this behaviour as making the eggs themselves more difficult to detect, rather than camouflaging the nest itself. This might be effective if the nature of the nests and the ground topography makes it more effective for (likely overflying avian) predators to cue on eggs rather than whole nests. Such a situation might apply here: the eggs contrast strongly against the background substrate and the nests are the relatively simple scrapes characteristic of many plovers. The authors could not demonstrate a link between any camouflage variable and the probability of nest predation – but predation rates were so high at their study site that any effects may have been masked by a ceiling effect. Partial covering of eggs only in response to imminent risk of predation has also been reported in the closely related white‐fronted plover (*Charadrius marginatus*: Summers & Hockey, 1981), where covering behaviour was unrelated to ambient or substrate temperatures, ruling out a primarily thermoregulatory function.

Amat, Monsa & Masero (2012) argued that the covering of eggs with nest material prior to parental departure from the nest shown by Kentish plovers (*Charadrius alexandrines*) serves both to enhance thermoregulation and to enhance camouflage by reducing the difference in coloration between eggs and the visual background provided by the nest. Opermanis (2004) similarly demonstrated that artificial duck nests without covering of eggs with nest lining were more heavily attacked by overflying avian predators. Prokop & Trnka (2011) and Keller (1989) reported the same in the floating vegetation‐constructed nests of little grebes (*Tachybaptus ruficollis*) and great crested grebes (*Podiceps cristatus*), respectively. Kreisinger & Albrecht (2008) found that artificial duck nests where the eggs had been covered with nest material were harder for humans to locate on photographs that nests with the eggs exposed.

It has also been speculated that the decorations added to some spiders' webs act to provide a background against which the spider is camouflaged from its predators and/or its prey [see Théry & Casas, 2009 for a thorough review]. The situation is complex because these decorations may also act to draw the attention of predators away from the spider and/or to entice prey towards the web – and there is empirical evidence for both of these functions in at least some species (Théry & Casas, 2009). It may be that the relative importance of different benefits varies with species or even with different ecological niches inhabited by different spiders of the same species. Gan *et al*. (2010) used careful modelling of visual systems to argue that decorations in their focal species *Cyclosa octuberculate* theoretically reduced the ability of both avian and wasp predators to detect the spider by separating it from the visual background. Demonstrating that such a camouflaging mechanism leads to reduced rates of attack by predators is challenging. Chou *et al*. (2005) manipulated the presence of decorations on the webs of *Cyclosa confusa* and found that decorations reduced the attack rates of wasps on the spiders. However the attack rates by wasps were higher for decorated webs, but many of those attacks were not directed at the spider but at the decorations – in this case it seems that the decorations act as a decoy rather than (or possibly as well as) camouflaging the spider. Gonzaga & Vasconcellos‐Neto (2005) explored this using artificial webs and model spiders based on the genus *Cyclosa*. They found reduced evidence of attacks on clay model spiders in artificial webs with decorations, and attributed this to decoration disrupting the appearance of the spider's outline (a camouflaging effect). Low levels of markings on the clay suggestive of attempted predation on the decorations argues against protection through decoying – but in the absence of observations of predators this conclusion has to be tentative. An interesting variant of this idea has been suggested in leaf beetles. Here, body coloration of various species may have evolved to resemble, or act as masquerade to, the damage on leaves (decoys) produced by beetles themselves as they feed (Konstantinov, Prathapan & Vencl, 2018).

In summary, it seems highly likely that a number of ground‐nesting birds have evolved specialist egg‐covering behaviours to enhance the camouflage of their eggs when unattended. Further it seems that this is often achieved through modification of the background against which the eggs are viewed so as to make detection more difficult. It remains possible that similar background matching to enhance crypsis occurs in web‐spinning spiders, but this has not been demonstrated conclusively. In this case the challenge to researchers is that aspects of web appearance can influence predators and prey of spiders in a complexity of ways other than by providing a visual background to the spiders – and so partitioning out any effect through background manipulation is more difficult.

### (3) Hiding built structures

The evidence that animals employ behaviour to hide the structures that they build is scarce. Almost by definition, traps such as spider's webs must be constructed in such a way as to be difficult for prey to detect in order to be effective, and there is evidence that certain specific features of webs can be linked to reduced avoidance by prey [see Théry & Casas, 2009 for a review]. Turning to homes built by animals, Bailey *et al*. (2014) provide evidence that birds may actively select materials that camouflage their nests. They demonstrate that captive zebra finches (*Taeniopygia guttata*) preferentially select nesting material that is similar in colour to the provided nest cup and surrounding cage walls. What we need next is a demonstration of similar selectivity by wild birds and an exploration of whether such selectivity affects nest predation by visually mediated predators. A considerable number of bird species collect lichen flakes, white silk cocoons and white‐coloured man‐made materials and incorporate them in a scattered effect across the outer surface of their nests. Hansell (1996) argues convincingly that a very plausible explanation for this is camouflaging the nest through disruption of its outline. We would welcome exploration of this idea with baited nests utilised after the fledging stage – randomised to a control or experimental treatment where such ‘decorations’ are carefully removed.

In summary, a range of studies suggest that birds my actively choose certain materials used in the construction of their nests in a way that enhances camouflage – but we currently lack evidence that demonstrates that such nesting material preferences actually reduce rates of nest discovery by visually orientated predators.

## VI. MOVEMENT

It is generally considered that stillness is an integral aspect to camouflage, and there are many reports of cryptic prey ‘freezing’ in response to heightened danger from visual predators (e.g. Caro, 2005). This is backed up by experimental demonstrations that movement by an organism with cryptic colour and patterning can greatly increase its chance of detection (e.g. Ioannou & Krause, 2009; Stevens *et al.,* 2011). Here we explore if there might be some exceptions to this generality.

### (1) Flicker‐fusion

The flicker‐fusion effect occurs if a patterned object moves sufficiently quickly across the visual field of a viewer that the patterning becomes blurred and the appearance of the patterned object changes (Endler, 1978; Stevens, 2007; Umeton, Read & Rowe, 2017). It is plausible that such an effect could reduce the predation risk of moving objects by providing a better match to their background, but while this has been postulated to occur in some fast‐moving striped snakes the evidence is scant. Allen *et al*. (2013) did find an association across snake species between longitudinal stripes and rapid escape speeds, but concluded that the flicker‐fusion hypothesis is theoretically possible but unlikely to occur in nature. Indeed, it would generally be expected to be linked with stripes orthogonal to the direction of motion. Titcomb, Kikuchi & Pfennig (2014) provided calculations to suggest that some snakes could in principle move fast enough for the flicker‐fusion effect to occur when their banded patterns were viewed by avian predators (particularly when viewed under low light levels). However, no data on critical flicker‐fusion thresholds for snake‐eating raptors are available, and the likelihood of flicker‐fusion camouflage working may depend on the predator keeping its head fixed rather than moving to track the prey. Lindell & Forsman (1996) demonstrated that males of the zigzag morph of the adder (*Vipera berus*) had higher survival than those of the melanistic morph (with the opposite effect being seen in females). These authors suggested that this is consistent with protection for the zigzag morph through flicker‐fusion in the faster‐moving males; however, we do not yet know if the difference in survival is even linked to predation, let alone the mechanism underlying any predatory mechanism. Other authors have argued that the zigzag pattern in vipers acts as an aposematic signal (e.g. Valkonen *et al.,* 2011). Thus, at present while it is theoretically possible that the flicker‐fusion effect could contribute to behaviourally modulated camouflage, there is very little evidence to implicate it in any biological system. Our belief is that a relatively specialised set of circumstances (e.g. background type, relative distance, speed and trajectory of target and observer, lack of other landmarks on the target for viewer to cue on) would be required for flicker‐fusion to contribute a strong selection pressure. Thus, this phenomenon may not have widespread relevance to camouflage in natural systems. Besides experimental work in natural systems, it would be valuable to undertake proof‐of‐concept work in artificial stimulus experiments (similar to motion dazzle, see Section VI.2) to determine the viability of this strategy, as well as obtaining more data on critical flicker‐fusion thresholds for predators to allow calculations of the appearance of moving prey.

### (2) Motion dazzle

Motion dazzle is the phenomenon where the pattern of a moving object can make estimation of its speed and/or trajectory harder (Thayer, 1909; Stevens, 2007; Stevens, Yule & Ruxton, 2008). Proof of concept in artificial systems has been repeatedly demonstrated for human observers (e.g. Scott‐Samuel *et al.,* 2011; Stevens et al., 2011, 2008, and references therein), but highly suggestive evidence also exists for non‐human observers (Santer, 2013; Hämäläinen *et al.,* 2015). Patterning that may cause a motion‐dazzle effect has been hypothesised for a number of animals (especially lizards: Halperin, Carmel & Hawlena, 2017; Murali & Kodandaramaiah, 2018), but has been investigated most fully in the case of zebra (*Equus* spp.) stripes (How & Zanker, 2014). However, from our perspective here it is important to emphasise that motion dazzle is not a form of crypsis; it may be a mechanism that acts to reduce the vulnerability of prey to predators, but not through reducing the likelihood of the predator detecting and identifying the prey. Rather if motion dazzle is effective it will be so after the predator has detected the prey, the prey is fleeing, and the markings of the prey reduce the probability that the predator will effectively track the movement of prey during pursuit and successfully turn pursuit into capture.

It is at least theoretically possible that the same markings could offer camouflage to stationary prey and a motion‐dazzle benefit after the prey is in flight. However, empirical evidence suggests that those markings that are effective for either of these mechanisms are not effective for the other (Stevens *et al.,* 2011). That said, work has so far been restricted to humans visually searching for and then tracking artificial prey on a computer screen, and there might be potential for markings to combine being effective *via* these two mechanisms in ecological situations where the two mechanisms operate at different spatial scales. It has been demonstrated that the striped markings of some fish allow them to combine signalling to nearby conspecifics with camouflage against predators that view them from a greater distance, because the stripes blend together visually when viewed from sufficiently far away (Marshall, 2000). However, the potential to combine dazzle and crypsis in this way is at present speculative. Finally, an intriguing experiment by Pike (2015) raises the possibility that iridescence (or interference) coloration, where perceived colour changes with angle of viewing, might function to reduce capture of moving prey. Pike presented captive birds with artificial moving prey on touch screens and found that, compared to control prey, iridescent targets incurred more pecks before capture and that missed pecks were more inaccurate, despite no difference in latency to attack between prey types. As yet, this idea has not been explored further but merits study. Finally, note that there may be interesting interactions between some camouflage types, motion dazzle, and other defences in group‐living animals, for example the confusion and dilution effect (see for example Hogan, Cuthill & Scott‐Samuel, 2016).

### (3) Motion to facilitate masquerade

Bian, Elgar & Peters (2016) present evidence that is suggestive that a stick insect (*Extatsoma tiaratum*) shows swaying behaviour in windy conditions in order to reduce the ease with which it can be distinguished visually from surrounding moving foliage. They found that the frequencies adopted by the insect overlapped strongly with those of the surrounding foliage. Further, they show that this is an environmentally sensitive behaviour – with swaying being more sustained in response to time‐varying (blustery) wind and ceasing at high wind‐speeds. This latter observation may be because swaying is too risky in terms of losing contact with the substrate in these conditions, or because the insect cannot produce movements of sufficient speed and amplitude to match the movement of vegetation in strong winds. While these results are highly suggestive of motion being used to facilitate crypsis and/or masquerade by stick insects, we await follow‐up studies that explore the effect of this on the detection of stick insects by ecologically relevant visual predators or prey.

Huveneers *et al*. (2015) report on the approach trajectories taken by white sharks (*Carcharodon carcharias*) attacking bait. They found that over the course of the day the sharks tended to change their approach direction so that the sun was often directly behind them; this non‐uniformity of attack direction was only seen on sunny days. It is not clear what the functional mechanism underlying this behaviour is, but it is plausible that they ‘attack out of the sun’ as a means of reducing the ease with which they can be detected by prey. If so this could be considered a behavioural modification to improve crypsis, although alternative or additional mechanisms might involve better ability of the sharks to track their prey visually.

In summary, the general view that stillness promotes crypsis seems to be well founded. However there may be some circumstances where movement of background features can be mimicked by an organism in a way that enhances crypsis. Alternatively, or additionally, it may be more difficult to detect a moving organism against some types of backgrounds (e.g. bright sunlight for sharks), and it is possible that some organisms have evolved to exploit this effect behaviourally. However investigations into these possibilities are just beginning. There has been more consideration of a link between flicker‐fusion and crypsis – but we have yet to find strong evidence for its relevance to any natural system. It seems more likely that some mobile animals use motion dazzle to mask their speed and direction, but so far the indications are that the traits that would confer such an advantage to moving animals might be antagonistic to crypsis when at rest.

## VII. ROLE OF BEHAVIOURAL CAMOUFLAGE IN A CHANGING WORLD

Humans are having a huge impact on other organisms globally, but these impacts are diverse and not always easy to predict. Here we provide case studies that argue for behaviourally mediated camouflage to be considered an important trait that can mediate the impact of humans on different animals in nature.

Behaviour may be a vital component of phenotypic plasticity as a means of ameliorating the negative impacts of climate change on camouflage. Obvious species to explore in this regard are those that show seasonal colour polyphenism associated with seasonally varying requirements of background‐matching camouflage. One such species is the snowshoe hare (*Lepus americanus*) that moults into a white winter coat and a grey‐brown summer pelage. Mills *et al*. (2013) reported that the hares display limited plasticity in response to timing of snow arrival and melt. It seems plausible that (in both spring and autumn) hares could preferentially feed in areas that offer the best camouflage for their current coat, and behaviourally become more wary of predators when they use habitat that does not match their current coat colour. As yet, there is no evidence that hares modify their behaviour to cope with mismatches and choose appropriate backgrounds (Zimova *et al.,* 2014). However, snowshoe hare are known to vary their behaviour to ameliorate enhanced predation risk associated with brighter moonlight (Griffin *et al.,* 2005) or foraging in gaps in the forest canopy (Hodson, Fortin & Bélanger, 2010), and so this merits further investigation. More generally, the role of behaviour in mediating the consequences of climate change for camouflaged organisms deserves urgent research effort.

Behavioural flexibility may also allow organisms to exploit novel environments produced by current global change associated with the increased human footprint on the planet. An interesting example of this is the positioning behaviour of grasshoppers colonising street pavements as a novel habitat (Baños‐Villalba *et al*., 2018; see Section III.1). These grasshoppers aligned themselves with the spaces between adjacent pavement bricks more often than would be expected by chance, especially so for individuals whose coloration did not allow a good match to the background provided by any part of the pavement. The authors interpreted this alignment behaviour as offering an anti‐predatory benefit *via* camouflage. Further they argued that this behavioural flexibility has been key to allowing the grasshoppers to exploit a novel man‐made habitat. We suspect that other examples of behaviourally modulated camouflage will be found in other species that increasingly exploit urban (and other man‐made) environments.

Finally it is possible that behavioural flexibility in camouflage may sometimes be an important trait facilitating invasion of natural habitats consequent to deliberate or inadvertent introduction by humans. The shore crab (*Carcinus maenas*) is known as a globally invasive species (Darling *et al.,* 2008), and several studies have shown phenotype–environment associations (e.g. Todd *et al.,* 2006; Stevens *et al*., 2014). Although work has yet to demonstrate conclusively that camouflage offering protection from visual predators is the driver of this phenotype–environment association, other possible explanations are less plausible, and matching is likely to arise *via* both colour change over the medium to long term (Stevens, 2016) and potentially to behavioural choices of substrates (Todd *et al.,* 2012; Nokelainen *et al.,* 2017). Species like this may be ideal candidates for the exploration of how novel environments can be rapidly and successfully colonised after an initial invasion.

## VIII. FUTURE WORK, KEY QUESTIONS, AND CONSIDERATIONS

Despite a wide range of research on various aspects of behaviour and camouflage, there are numerous areas requiring further consideration. We provide some suggestions for future research above, and highlight seven of the most important questions below.

(1) *How widespread is behavioural choice of substrate and position in individual animals?* It is clear that many animals select backgrounds for concealment, but many studies do not distinguish between species‐level and individual‐level choice, and work on the latter is restricted to only a few groups.

(2) *What is the significance of variation in individual substrate choice?* There is often considerable variation within and among individuals and morphs in the choices made of substrate (or in the presence of choice). However, why this exists, and what significance it has is unclear.

(3) *What are the mechanisms that control choice of substrate at species, morph, and individual level?* There is some evidence both for a genetic role and for comparisons of appearance with the background, but explorations have been very limited to date and almost no work has been invested on areas, such as imprinting. Work on the sensory modalities used is also sparse. Currently, there have been only a handful of studies directly testing the role of how sensory information is used, including visual and mechanical information. Understanding the mechanisms involved is likely to have value for understanding aspects of behaviour more widely.

(4) *How do animals use behaviour to prevent the production of give‐away features, such as shadows?* Behaviour is likely to be important for strategies like camouflage *via* countershading (e.g. Penacchio *et al.,* 2015) and to hide the presence of shadows cast on the ground, yet remains almost unexplored in natural systems.

(5) *How widespread is modification of the visual background, and what advantage does this bring?* There are suggestions that a range of animals modify the environment around themselves, their nests and other structures to reduce detection; but experimental work is needed, including assessments of its adaptive value.

(6) *How key is movement to the success of some camouflage strategies, and how ecologically plausible are they?* Movement has been suggested to be important in some forms of masquerade, yet has rarely been tested. In addition, movement is an important aspect of both flicker‐fusion camouflage and motion dazzle, yet ecologically relevant experimental work is scarce.

(7) *What is the role of behavioural camouflage in a changing world?* Plasticity in behaviour and strategies that enable animals to cope with rapidly changing environments may help them to cope with environmental change, including the maintenance of camouflage.

## IX. CONCLUSIONS

(1) Camouflage has long been known to be mediated by behaviour, including potentially in the choice of appropriate resting backgrounds, body positions and orientations, hiding key features such as shadows, maintaining concealment during motion, and modifying bodies, structures, and the surroundings.

(2) Evidence for animals choosing appropriate substrates and positions is convincing at species, morph, and individual levels. However, how widespread this is across taxa is not yet clear, with most work focussing on birds and moths, and a few other groups. In addition, multiple mechanisms may be in operation, including imprinting, genetic links between appearance and behaviour, and comparisons of appearance to the substrate. At present, while some evidence exists for different mechanisms among species, it is limited in both scope and rigour. The ecological significance of choice in habitat use and niche partitioning for camouflage has yet to be properly investigated.

(3) Animals likely use behaviour in addition to countershading coloration to hide shadows cast on their bodies and the ground, but further experimental exploration of this is needed.

(4) It seems clear that a wide range of species, especially crabs and insect larvae, use decorating behaviour to modify their appearance for camouflage. However, direct experimental proof is often lacking and experiments are needed to determine the adaptive value of such behaviour. Other animals also seem to modify the visual environment (e.g. nest structures or nesting areas) for concealment.

(5) Movement is thought to be a key aspect of several camouflage strategies, including flicker‐fusion camouflage and motion dazzle. However, most work has been either restricted to indirect comparative studies of coloration (in flicker‐fusion systems), and experiments with artificial systems and human ‘predators’ (in motion‐dazzle studies). Additional experimental tests in more ecologically relevant systems are needed to establish the feasibility of these strategies. Motion is also likely key to some forms of masquerade, such as mimicking wind‐induced movement.

(6) Behavioural modification of camouflage may be an important route *via* which animals can cope with a changing world, and the significance of this needs urgent attention.
